# Immigrant parents’ experience with the Swedish child health care system: A qualitative study

**DOI:** 10.1186/s12875-017-0604-6

**Published:** 2017-03-01

**Authors:** Elisabeth Mangrio, Karin Persson

**Affiliations:** Department of Care Science, Faculty Health and Society, Malmö University, Jan Waldenströms gata 25, 205 06 Malmö, Sweden

**Keywords:** Migration, Qualitative research, Child health care, Support

## Abstract

**Background:**

Immigration, particularly when it is involuntary, is known to be an emotional stressor, regardless of the reason behind it. It is always a challenge to be removed from the habitual and cultural action pattern of the person or family. This can make children more vulnerable, because they often arrive with an increased risk of poor physical health. Because of that, it is crucial that immigrant children have access to ongoing health care. The aim of this study is to shed light on the experience of non-European immigrants with Sweden’s Child Health Care system.

**Methods:**

Qualitative semi-structured interviews were conducted, with parents of children who were patients of one of the four child health care centres. The centres were in four areas in a town in southern Sweden in which there are substantial immigrant populations. The interviews were conducted, transcribed and then analyzed with content analysis.

**Results:**

The results were divided into two main categories: The first is “the sense of being cared for in another way,” which was divided into the following four subcategories: compare with the home country, getting a home visit, engagement and contentment and unfamiliarity with the language. The second main category. “The feeling of getting all the practical needs met through the child health care system” had the following four subcategories: The importance of advice and guidance, getting oral and written information, getting help when needed and getting support when needed.

**Conclusions:**

The parents expressed contentment regarding the Swedish child health care and they were thankful for how it was organized, the engagement of the nurses, the information and advices given as well as for the opportunities of getting a home visit after birth. However, more research is needed in order to find out the extent to which the Swedish child health care system is culturally appropriate in the whole country.

**Electronic supplementary material:**

The online version of this article (doi:10.1186/s12875-017-0604-6) contains supplementary material, which is available to authorized users.

## Background

Immigration, particularly when it is involuntary, is known to be an emotional stressor [1]. Refugees have generally experienced high-stress conditions before the exodus, and the exodus itself may have been perilous. The arrival in the new country is usually followed by the asylum process, which is a time of uncertainty and possibly anxiety that can intensify any existing mental illness [1, 2]. Newly arrived families often settle in neighbourhoods with lower socioeconomic status; they often work in jobs with poor pay; and they may experience societal discrimination [1, 3, 4]. According to the qualitative study by Samarasinghe et al, the authors concluded that the stress parents experience during the residence-permission process is often transferred to their children and, as a result, the youngsters experience symptoms of stress [5].

Regardless of the reason behind the immigration, the absence of the person or family’s cultural patterns—customs, values and attitudes toward males and females and the infirm and the healthy, ways to express feelings and solve problems—is a challenge. Learning a new culture and language patterns is gradual, and throughout process, recent immigrants can experience significant stress, both physically and mentally [1, 6].

Immigrant children are not immune to the stress and, in fact, these circumstances can make children suffer from poor physical and mental health [6, 7]. According to studies by Hjern et al [8] and Schiariti [7], immigrant children need special attention since they have an increased likelihood of suffering from poor physical health. Special attention must be directed toward the lower breastfeeding rates among immigrant mothers, as well toward nutritional deficiencies among their children [8]. Earlier research has reported that migrant children face an increased risk of infections, behavioural problems, sleep problems and intentional and unintentional injuries. Immunisations are often delayed and hearing and vision and dental problems of many migrant children are neither assessed or treated [7]. It has been found that 40 to 50% of these youngsters suffer from psychiatric and psychosomatic disorders; this is mostly attributable to having been uprooted [8]. Therefore, it is crucial that these children receive social support because of the increased likelihood to suffer mental illness [8]. Providing appropriate services to migrant children will protect their physical health and their psychological and emotional well-being [7]. Since many immigrant children have been raised in societies where living conditions and child health care programmes differ considerably from those in Europe, these differences should be addressed in a structured way through the child health care program as soon as the children arrive in a new country [8].

A study that focused on primary health-care nurses’ impressions of health among families who immigrated involuntarily revealed that nurses encountered family members suffering from depression and physical conditions such as eczema, gastritis and generalized body pain [9]. The nurses also noticed that the stress caused by the process of immigrating worsened the conditions of immigrants with chronic diseases such as diabetes and asthma. And as time passed, the women in particular were perceived to be feeling isolated and experiencing stress due to the unfamiliar demands and responsibilities. They suffered from tiredness, homesickness and resultant somatic symptoms [9].

Negative attitudes toward immigrants hindered the families from assimilating into society, and unemployment and inactivity intensified the situation and resulted in diminishing self-esteem [5]. Conflicts within immigrant families may occur because of the challenges parents face in adjusting to their roles in a new environment. A father who is unable to find employment may feel like a poor role model for the children, and this can lead to violent behaviour within the family. Language barriers often force children to act as translators for the parents and, this too, can make the parents feel inferior. It must be noted that a family’s socio-economic background plays an important part in the family’s ability to adapt to the new country; well-educated parents have been shown to adjust more easily than do parents with limited education [5].

In Sweden, 20% of all children have either one or two parents who were born abroad [10]. These children have an increased risk of ill health, usually in the form of poor dental health and greater likelihood of being injured at home or in the surrounding environment. Furthermore, the very act of uprooting children from their home countries can not only affect their health and well-being, but it can also negatively impact their family members [8].

Most of these families are in contact with Sweden’s Child Health Care system [11]. Sweden has a tax-financed health system, and municipalities are responsible for providing general access to health care [12]. All children residing in Sweden legally are eligible, without charge, for CHC services and CHC is a well-established organisation with responsibility for reducing morbidity, mortality and disability in newborn and younger children. Through regular visits to the CHC, each child’s weight and physical and developmental health are closely followed and vaccinations are given until the child is 5 years. Another purpose is to educate parents so that they can make the most of their child’s developmental opportunities and that is done through parental groups offered during the child’s first year. For all immigrants with low knowledge and fluency in Swedish, a translator is booked for the visits at CHC. Written information that is distributed at CHC is translated into the most common languages that are represented in Sweden [11].

Studies have shown health care professionals confront many challenges in working with immigrant families. In their work with immigrant children in the CHC, primary health nurses said they lack written guidance, emotional support and help with the workload. A majority of the nurses experienced difficulties in their interactions with children and parents of foreign origin, and a majority also said they lacked formal training in cultural competence [9].

Addressing children’s health problems is mostly regarded as a difficult matter among nurses, especially if the nurse and parents do not share the same language and an interpreter is not available [9, 13]. According to Woodland et al [13], it is of great importance that the nurses at the CHC interact with immigrant families with cultural sensitivity and that they tailor the information provided to parents and children to facilitate comprehension. Therefore, there is a need to examine the child health-care-provision experience that immigrant parents encounter in Sweden from the CHC service. To the best of our knowledge, no previous studies have been conducted on this topic as it relates to services received by families from outside of Europe.

## Methods

### Design

A qualitative method was used, in which semi-structured interviews were conducted with parents of children attending any of the four participating CHC centres in a town in southern Sweden. The interviews were conducted, transcribed and then analysed with content analysis [14]. The first author (EM) has several years of working within a multicultural area within the field of child health care and have done previous research with populations of different nationalities.

### Sampling frame

The sample consisted of 19 parents (14 females and five males) who, during the data-collection period, sought treatment for a child at any of the four participating CHC centre in southern Sweden. Families in which both parents immigrated from non-European countries and whose child was under the age of 5 years old met the sample criteria. Participating parents, who ranged in age from 21 years old to 45 years old were also required to either speak understandable Swedish or English to be included in the sample. The length of residency in Sweden for these families ranged from 2 years to 22 years.

### Data collection

In the pre-data collection period, the chief administrators of 27 CHC centres were contacted by email to inform them of our study. Six showed an interest in taking part in the actual study. However, when registered nurses at these centres were asked about their willingness to act as gatekeepers, only one CHC was willing to participate. Therefore, the email canvassing process began anew in the same town, resulting in another three CHC centres becoming participating sites, because nurses from each were willing to participate as gatekeepers. Thereafter, continual contact was maintained with the gatekeepers, and they contacted the first author (EM) whenever eligible families were at the centres.

All interviews were conducted by the first author (EM) and the interviews were done either at a CHC facility or at the homes of participating families. An interview guide was used, see Additional file 1 and interviews were tape-recorded and transcribed directly after the interviews. After the pilot interview was completed, the second author (KP) read the interview and provided suggestions about ways to improve subsequent interviews. The interviews were conducted in Swedish (*n* = 15) and in English (*n* = 2). The interviews lasted between 5 and 15 min, with an average duration of 9,5 min per interview. Participating parents’ countries of origin as well as the number of parents from each are shown in Table 1.

**Table 1:** Number of participants and country of origin

Countries of origin	Number of participants
Afghanistan	2
Chile	1
India	1
Iraq	4
Kurdistan	3
Kuwait	1
Lebanon	1
Pakistan	2
Palestine	1
Venezuela	1
Vietnam	2

### Data analysis

Interviews were transcribed verbatim and checked against the original audio recording. The coding of the analysis was done by the first author, and the second author commented on the analysis. The interviews were analysed using content analysis with the purpose of identifying emerging themes and patterns, according to Burnard [14]. The analysis process was as follows:

1. Interviews were read and transcribed verbatim in either English or Swedish.
2. Open coding was performed, and used to create categories.
3. Matching subcategories were grouped into new categories.
4. The results were discussed by the authors, and adjustments were made continuously until all data were used.

When no data was suitable for more than one category, the process was considered complete. Then the titles of the categories and subcategories were evaluated to ensure that they provided a clear picture of the actual content. Table 2 provides excerpts (meaning units) from the interviews and the resultant, code, subcategory and category.

**Table 2:** Some extracts from the interviews, explaining the analysis through meaning unit-category

Meaning unit	Code	Category	Sub Category
We didn’t know about the vaccination schedule, since it is different from India. The nurse helped us a lot, giving us information about all the different vaccinations that should be given to the kids, and she had a regular follow- up as well, so that was very good for us.	Didn’t know about vaccinations and the nurse gave us information.	The feeling of getting all the practical needs met through the child health care system	The importance of advices and guidance
If I don’t understand something, I tell the nurse and then she can explain for me, and explain if I misunderstood something. I am very content.	They explain for me when I don’t understand.	The sense of being taken care of in another way	To not know the language

## Results

After analysis, the results were divided into two categories: 1) The sense of being cared for in another way and 2) The feeling that all the practical needs were met through the child health care system.

### The sense of being cared for in another way

This overarching category emerged from many of the parents who mentioned that when they compared Swedish child health care provision with that of their countries of origin, they felt gratitude and thankfulness. They mentioned appreciation for the CHC system, the way the service was organized and how well children were cared for. Appreciation was also expressed for the interest that nurses showed for the children and that the nurses found ways to communicate with parents despite language differences and challenges. The following sub-categories emerged from this category: *comparison with the home country, having a home visit, engagement and contentment*, *unfamiliarity with the language.*

#### Comparison with the home country

Several interviewees expressed that the Swedish child health care system was good and that no similar health care could be found in their home countries. Some of them spoke about the lack of knowledge among women in their home countries regarding how to care for themselves and a newborn. They also mentioned equity in treatment compared with their home countries. They thought it was very good to have access to the Swedish CHC.

*In Sweden, it is not like in our home country. It is not unpleasant and terrible like it is in my home country.* -Informant 5

Another interviewee expressed that hygiene, engagement and treatment from nurses in Sweden was better than that in the home county. A participant expressed the difference like this:

*In my home country, the health care you get depends on money and which doctor you have and if you are poor you don’t get the same treatment as the rest. It is better for my children to grow up here in Sweden. -*Informant 6

All parents interviewed had similar thoughts about the CHC; one parent expressed it like this:

*There is a great difference here compared to my home country, and I am very content here. It wouldn’t have been the same health care in my country.* -Informant 15

##### Having a home visit

Getting a home visit from a nurse after delivery of a newborn was something that all interviewees expressed as quite different than their experiences in their home countries. Several expressed gratitude and appreciation for the home visits by the visiting nurse after their child’s birth. They felt honoured and as though they were important persons when someone from the health care system took their time to visit in their home. Parents expressed that they appreciated the chance to sit down for a while and talk to the nurse during the visit. One of the interviewees expressed it this way:

*It feels unbelievable! It is not like that in our home country and I felt so much of respect from the nurse when she came to our place. The nurses respect that a woman after birth feels tired and therefore they come to your place*. -Informant 9

Three participants expressed that they would have liked to have gotten a home visit, but it did not materialize. One woman mentioned that she had an appointment booked twice for a home visit, but it was cancelled each time, and she eventually needed to go to the CHC instead. Another mother said that she had not had any home visits for either of her two children and that she wished she would have gotten one. The third woman had enjoyed home visits when her first child was born, but the home visit for her second child was cancelled, and she felt disappointed.

#### Engagement and contentment

Almost every parent interviewed said that they were content with the level of involvement that the nurses exhibited when they visited a CHC centre. There was great appreciation for the nurses, who were described as nice, happy, and engaged with the families. The parents felt safe with the nurses and felt included while meeting with the nurses. Parents who brought their children to a CHC facility in a multicultural area characterized the entire staff as very nice. They attributed this ecumenical concern to the nurses’ meeting people from many different cultures and that it created good encounters for them with the nurses. A mother spoke about their satisfying encounters with nurses like this:

*You feel welcomed by the staff and we like especially the staff nurse in the waiting room and our own pediatric nurse. It is nice here and there is a warm atmosphere here*. - Informant 8

A few parents expressed that they were not as content as the interviewees above seemed to indicate. Some said that they would prefer to be reassigned to another nurse. In addition, another parent voiced contentment but mentioned that other parents not included in this study, were not as pleased. Some parents appreciated the doctors’ visits that their children had, and they characterised the doctors as nice. Some parents believed that the nurses had genuine concern for their children and exhibited thoughtfulness about the children. And other parents mentioned that they believed that the CHC medical professionals respected their cultural background, and they had not experienced racism.

#### Unfamiliarity with the language

One mother mentioned her appreciation that the nurses explained and adjusted their language as soon as she told them that she didn’t understand. However, she felt concern for the mothers with newborns if their Swedish-language proficiency was not as good as her own. Another mother explained that in the beginning, her Swedish was not good and, during that time, her interactions with nurses had not been as good as they were at the time of the interview. Other women did not get on well in the parental group meetings, and mothers had a difficult time understanding one other.

One father expressed the difficulties with language like this:

*My wife gets a translator as soon as I am working, and through that way she gets a lot of information and understands.* -Informant 14

Other parents also got translation assistance and appreciated it. One father, whose experience of getting language help was satisfactory, expressed it like this:

*Yes, my nurse came to our house and he is good and he talked in my language and first he called us and then we met at our house and he explained all the coming visits at CHC during the coming years*. -Informant 16

### The feeling of getting all the practical needs met through the child health care system

This overarching category emerged from parents speaking about practical issues of child health care and that advice, guidance and information was provided to them when needed. The parents also expressed gratitude for practical help with referrals to other health care professionals as well as the support they got in their parenting roles, though some wished there had been more support and practical help available. From this, the following sub-categories emerged: *the importance of advice and guidance, getting oral and written information, getting help when needed, getting support when needed.*

#### The importance of advice and guidance

Several of the parents mentioned that they got advice about vaccinations, breastfeeding, growth, nutrition, child safety, as well as advice related to medical situations like as infections and diseases. One mother said the following:

*She was having a bowel movement, and I didn’t know that she would get that. It was happening every five minutes and we were wondering what it was and I went and asked my nurse and she gave me a prescription that could be got from the pharmacy. I didn’t know where to go and whom to ask but she helped me.* Informant 1

One parent got advice at the parental group about nutrition and sleeping patterns:

*They told us about nutrition at the parental group and that we could divide the portions six times a day and we have now tried that and the baby is sleeping much better after that. -*Informant 7

Several of the mothers and fathers thought that the nurses were good about giving proper advice and guidance, and when the nurses did not know what advice to give, they asked colleagues and came back to them with the information. Two parents mentioned disappointments due to having gotten wrong advice when seeking guidance on several topics.

#### Getting oral and written information

Several of the interviewed parents were content with the written and oral information given at the CHC facilities. Parents appreciated being told about information on the Internet as well as handouts distributed at the centre. Other parents were content with materials mailed to them. One mother said this:

*It has been very good, and we got letters home prior to every visit, and the nurse always gives oral information about the coming visit and what to expect, for example if there is going to be vaccinations given to the child* -Informant 4

Another parent appreciated that they had communicated orally with centre personnel prior to vaccinations and, therefore, they knew what to expect and how it would be done. This type of information calmed them.

One mother who had recently arrived in Sweden mentioned that she would have liked more oral information about how the health care system works in Sweden and where to go if the child gets sick. She was also uncertain about the offerings of the various health care sectors in Sweden and did not know if other services were available for her and her child. Another parent would have liked more information about, for example, parental groups that could be joined, and one parent would have liked more oral information about food and proper nutrition:

*The information about food and when to start giving different kind of food to the baby, that they could have explained a little bit more. They only gave a book and thought that I would read it. But I don’t think all mothers read the book, so it would have been good with more oral information about nutrition.* -Informant 10

Another parent who had just recently arrived in Sweden was very content with the information the nurse had told him and his wife. The nurse helped them understand about children’s sleeping patterns and nutrition. They believed they needed the guidance, and they appreciated it.

Some of the parents in the sample mentioned that they appreciated help and guidance they received on a variety of childcare topics such as required vaccinations, check-ups and practical help with referring the child to other health services when needed. One parent expressed:

*We have hearing deficit in the family and we were afraid that our child would have it as well and therefore we got help from the nurse to book an appointment for our child* -Informant 11

Another mother expressed gratitude about the practical assistance provided her when the CHC referred her child for a developmental assessment, because the child was not doing well on age-appropriate cognitive testing at four-years-old. The referral revealed that the child was in need of extra support from the rehabilitation. The mother expressed gratitude that the nurse that was observant about the child’s need for support and additional instruction or therapy. Another mother explained about the help that she got as a result of a social services referral:

*They have a social worker here at the Family centre and I have got a lot of help from her. She helps me and explain for me how I should handle my child in the best and most proper way.* -Informant 13

Another mother expressed feelings of thankfulness for the all help she gets:

*Everyone here knows me well and they are fantastic people. I feel that I get all the help that I need and always gets it when I need and ask for it*. -Informant 2

But a father expressed that, compared to 15 years before when he brought his now-older children to the centre, the CHC seems less helpful. The father said that he would have liked for young his children to be getting more check-ups and help than they are currently receiving. In a somewhat similar vein, one mother said that she would have liked a greater level of support with her second child, as she believed that she had received more with her first child.

However, this same mother was grateful for help she had gotten when the child had atopic eczema, and they needed to go to the emergency room. The nurse wrote a referral note which was accepted by the ER, and she was very thankful for that help.

#### Getting support when needed

Many of the study parents expressed gratitude for the nurses’ support. One mother who had not been in Sweden very long said the support she got from her nurse was fantastic and that it was needed. Another woman explained that she didn’t feel well after giving birth to her child, and she felt that the nurse was a great support. She continued by saying that she can visit or call the nurse whenever needed and that it is difficult to find such good support elsewhere. Another woman spoke of the support she is getting like this:

*My nurse expressed that I could talk to her if needed, and since I got postnatal depression, I came to her and talked, and she said that it was normal to feel like that and that felt good to hear.* -Informant 3

Other parents mentioned that the nurses were very supportive when they did not know what to do with their child. One parent expressed that when she became worried about a matter and told the nurse, the nurse listened with such interest that that she began to worry less.

Another mother expressed following support:

*They always ask how I feel as a mother, and that feels good.* -Informant 12

Another mother felt good about getting support from the nurse through the Edinburgh Postnatal Depression Scale (EPDS) screening that took place at when her child was 2 months old. She stated that it felt pleasant as a mother to come for that meeting.

## Discussion

The results showed that immigrant parents appreciated several components of the CHC’s service. First, the parents were grateful for the Swedish health care system when comparing it to the health care that would have been given them in their home countries and, although some parents had had difficulties understanding Swedish in the beginning, they appreciated the availability of interpreters. Also appreciated was that nurses tried to speak with the parents in their native language whenever possible. They also mentioned appreciation of the practical help they got through advice and guidance, in both written and oral form, concerning different facets of child care and parenting. The parents also reported that they were satisfied with getting practical help when they needed it in general.

Parents who had home visits seemed to appreciate them as a first encounter, and the parents who did not have one were disappointed. Those who did have a home visit explained that it was nice to be able to sit down with the nurse, especially after giving birth and feeling tired. In addition, some were afraid of going out with the baby due to infection risks. When comparing Sweden’s CHC to the health care their children would have received in their countries of origin, most agreed that they would not have gotten such visits there. A systematic review by Peacock et al [15] showed that home visits are important after birth from many standpoints: preventing illness, preventing a child’s growth failure, providing parental support and preventing child abuse. Oddly, a few parents in the present study did not have any home visits, despite the directive that every CHC centre in Sweden must provide such visits [16]. This indicates that resources should perhaps be reallocated to ensure that all required home visits are carried out.

Many of the interviewed parents said that they were content and grateful for the encounters with the nurses and thought that they were engaged and showed respect for the families. They were also thankful for translators and, when comparing CHC services with the health care in their home countries, Sweden was well-rated. According to an Australian study, health care services that are sensitive to their clients’ cultural and linguistic backgrounds are more likely to improve access and equity, health literacy and quality of care for immigrant children [13]. When health assessments are done with an appreciation for the client’s culture, it can provide useful insights into cultural, dietary and health practices. This is also in line with findings of Shen et al. [17], who state that cultural competence is an essential component for providing relevant and culturally responsive health care to diverse populations. This type of cultural sensitivity can help reduce ethnic disparities in health care provision and potentially enhance the quality of care, patient satisfaction and health outcomes [17]. Health care professionals must take into account that refugees leave behind culture, language, environment, climate, family, friends, social system and norms of behaviour [18]. Another important part of this equation is availability of translators for newly arrived refugees, which is simply good practice in health care [13], and several of the parents interviewed noted its utility. The overarching theme “the sense of being taken care of in another way” was emerged from comments about the engagement of the nurses that was good rated according to the interviewees and was different from the one they remembered in the home country. It could probably be the cultural sensitivity that was evident for these families that Shen et al is mentioning [17], as well as the fact that the CHC offers good service when it comes to translation. This translation service is probably not so common in the home countries and was appreciated because of that. According to a systematic review by Berendes et al [19], the authors concluded that both private and public health care in low- and middle-income countries had poor quality in their provision, but the private had a better care when it comes to responsiveness, effort and being client oriented. An additional reason might be that Swedish child health care is free of charge [11]. Another reason that could have influenced the families’ perceptions of the care given is that all children are offered the same national programme no matter where they come from [11]. Probably families comparing the Swedish child health care with that from the home countries could be related to these kind of aspects. All of this needs to be considered when working to improve the child health care system in Sweden.

Some of the parents in the sample expressed that they had gotten good support from the nurses regarding different matters, such as not feeling well after childbirth, especially after arriving in a new country and not knowing how to parent. As Samarasinghe et al mentioned, immigrant parents and their children experience stress in the new environment and, therefore, the support that the nurses provide can be essential to the families during this period in their lives [1, 5], especially because both parents and children may have experienced mental and physical challenges, and they typically need extra attention from health professionals [8].

The aim of the study was to cast light on the experience of non-European immigrant families with Sweden’s the Child Health Care system. The participating CHC centres were in a multiethnic town in the southern Sweden and, although chief administrators of all 27 centres in this town were contacted by email, only four administrators responded. No explanations were given for the low response rate, but it is likely that it is due to a high workload. The four CHC centres were located in areas with substantial immigrant populations, so we had a good representation for the purposes of this study. However, a greater segment of the population could have been included if translators had been more available to us. Another limitation of this study is that the interviews were only conducted in Swedish or English and that could have affected the result of the present study. It could be assumed that newly arrived parents with greater health care need and greater need for support were excluded from the present study because of language barrier. If a translator was available, the result could therefore have been more nuance. However, there were a few parents with shorter stay in Sweden included in the present study.

The result that emerged in the present study was mainly positive comments about the CHC service and it must be considered that there is a possibility that the parents felt that is was difficult to share negative experiences during the interviews. Although the first author mentioned before every interview that all the information from the families should not be spread to the CHC service.

The interviews were conducted by the first author (EM), and after the pilot interview was completed, the second author (KP) read the interview and provided suggestions about ways to improve subsequent interviews. The coding of the analysis was done by the first author, and the second author commented on the analysis, thereby increasing the credibility of the results because the perceptions of both researchers were taken into consideration and were thoroughly processed [20].

The transferability of a qualitative study can be suggested, but in the end, this is something for individuals to decide for themselves [20]. Several findings in the data were recurring, which suggests that they may be transferred to comparable situations.

## Conclusions

Non-European immigrant parents in Sweden expressed gratitude about receiving health care in a different way than they would have in their home countries. They were also grateful about the practical help they were afforded. Regarding childrearing, although the parents in this study seemed to get culturally sensitive and competent care, more research is needed in order to find out to what extent the Swedish child health care system is culturally appropriate to all peoples throughout the country.

## Additional file

Additional file 1: Interview guide. (DOCX 12 kb)

## Abbreviations

CHC: Child health care
EM: Elisabeth Mangrio
EPDS: Edinburgh Postpartum Depression Screening
KP: Karin Persson
