# Supplementary material for: Immigrant parents’ experience with the Swedish child health care system: A qualitative study
Source: BMC Fam Pract. 2017 Mar 1;18:32. doi: 10.1186/s12875-017-0604-6 (PMC5333410; doi:10.1186/s12875-017-0604-6)
Supplement: Additional file 1: — Interview guide. (DOCX 12 kb) [file 12875_2017_604_MOESM1_ESM.docx]

# INTErview guide

1. Could you please tell me about your encounters with the child health care?
2. What have you experienced as positive?
3. What have you experienced as negative?
